# Serum Zinc Concentration in Relation to Asthma Control and Treatment-Step-Based Severity in Adults: A Retrospective Cross-Sectional Study

**DOI:** 10.3390/diagnostics16213673

**Published:** 2026-11-06

**Authors:** Raziye Tülümen Öztürk, Hafize Titiz Yılmaztepe, Özge Aslantekin Eken, Burcu Çoban, Dane Ediger

**Affiliations:** Division of Allergy and Clinical Immunology, Department of Pulmonary Diseases, Faculty of Medicine, Bursa Uludağ University, 16059 Bursa, Türkiye; drhtitiz@gmail.com (H.T.Y.); aslantekinozge@gmail.com (Ö.A.E.); drburcucoban@hotmail.com (B.Ç.); ediger@uludag.edu.tr (D.E.)

**Keywords:** zinc, adult asthma, asthma severity, symptom control, trace elements, airway inflammation

## Abstract

**Background**: Zinc supports immune homeostasis and participates in antioxidant defense and the regulation of inflammatory responses. Lower circulating zinc has been reported in asthma, mostly in children; its relation to disease control in adults is unclear. **Objective**: We examined serum zinc concentrations in relation to symptom control and treatment-defined disease severity in adults with asthma and whether any association with control was independent of potential confounders. **Methods**: In this retrospective cross-sectional study of 145 adults with asthma, serum zinc was compared between controlled (Asthma Control Test [ACT] > 19) and uncontrolled asthma and across treatment-step categories, using regression models adjusted for age, sex, C-reactive protein (CRP), smoking, and treatment step. **Results**: In the crude analysis, zinc was higher in controlled than in uncontrolled asthma (mean difference 6.7 µg/dL, 95% confidence interval [CI] 1.0 to 12.3), and zinc < 70 µg/dL was more frequent in uncontrolled asthma (18.6% vs. 5.8%). After full adjustment, these associations were attenuated and no longer statistically significant (mean difference 3.1 µg/dL, 95% CI −2.6 to 8.8). Zinc was lower at higher treatment steps in the unadjusted comparison (*p* = 0.0497), but this difference was not significant after adjustment for age (*p* = 0.277), and exploratory comparisons were imprecise. **Conclusions**: Higher serum zinc was associated with controlled asthma only in unadjusted analyses. These findings do not establish an independent or causal relationship, and whether zinc supplementation improves asthma control requires further study.

## 1. Introduction

Asthma is a heterogeneous airway disorder in which variable respiratory symptoms and expiratory airflow limitation occur in the context of chronic inflammation [1]. Clinical assessment distinguishes current disease control from underlying asthma severity, although the two domains are related. Control encompasses recent symptoms and future risk, whereas severity is assigned retrospectively according to the treatment intensity needed to maintain control [1].

Asthmatic airway inflammation may disturb the balance between reactive oxygen and nitrogen species and endogenous antioxidant defenses [2]. An excessive oxidative burden may sustain inflammatory signaling and impair epithelial barrier integrity [2]. This has prompted interest in micronutrients that contribute to antioxidant protection and immune regulation in asthma [3]. Zinc is an essential trace element incorporated into numerous enzymes, proteins, and transcription factors. Its biological functions include epithelial maintenance, tissue repair, antioxidant activity, and modulation of innate and adaptive immunity [3,4,5,6]. Zinc status and inflammation may influence one another. Low zinc availability may compromise antioxidant and immune responses, whereas inflammation may itself change circulating zinc concentrations [6,7]. Circulating zinc concentration should not, however, be equated with whole-body zinc status. Serum or plasma zinc is the most widely used biomarker, yet it reflects only a small fraction of total body zinc and responds to inflammation, infection, fasting, and the time of sampling independently of dietary intake [7]. A low value measured during inflammation may therefore indicate redistribution rather than true deficiency.

Clinical studies of circulating zinc in asthma have produced inconsistent findings. Several observational studies and pooled analyses have reported lower serum zinc in people with asthma than in healthy comparators [8,9,10]. In a meta-analysis, this difference was evident in adult but not in pediatric studies; however, adult studies have mostly compared patients with asthma with healthy controls rather than examining zinc in relation to disease control [9]. In a pediatric study, higher serum zinc concentrations were found to be associated with better symptom control [11]. However, this relationship is not consistent across all studies. Differences in study populations, clinical characteristics, sampling conditions, and laboratory methods may contribute to the heterogeneity between the results [9,10,12]. Most clinical evidence comes from pediatric populations; corresponding adult data linking serum zinc with control and treatment requirements remain limited. In adults, it is still unclear whether serum zinc tracks current symptom control, the treatment intensity needed to achieve it, or both, and whether any such relationship survives adjustment for age, sex, and systemic inflammation, each of which influences circulating zinc in its own right [7]. Because control captures recent symptoms while treatment step reflects longer-term therapeutic need, examining the two separately may help clarify which aspect of the disease, if either, is related to zinc.

We therefore evaluated the association of serum zinc with asthma control in adults, examined whether this association was independent of potential confounders, and compared zinc concentrations across severity categories defined by treatment requirements. We also examined zinc in relation to asthma-related hospitalization, emergency department attendance, systemic corticosteroid exposure, and biologic treatment.

## 2. Materials and Methods

### 2.1. Study Design and Population

We conducted a retrospective cross-sectional analysis of adults evaluated for asthma at the Adult Allergy and Clinical Immunology Outpatient Clinic of Bursa Uludağ University Faculty of Medicine, a tertiary referral center in Bursa, Türkiye, from January 2022 through January 2024. Eligibility required an age of at least 18 years and a physician-confirmed asthma diagnosis consistent with Global Initiative for Asthma recommendations [1]. Only patients with a positive bronchodilator reversibility test documented in their records were included. The institution’s electronic records served as the source of clinical and laboratory information.

**Figure 1:**
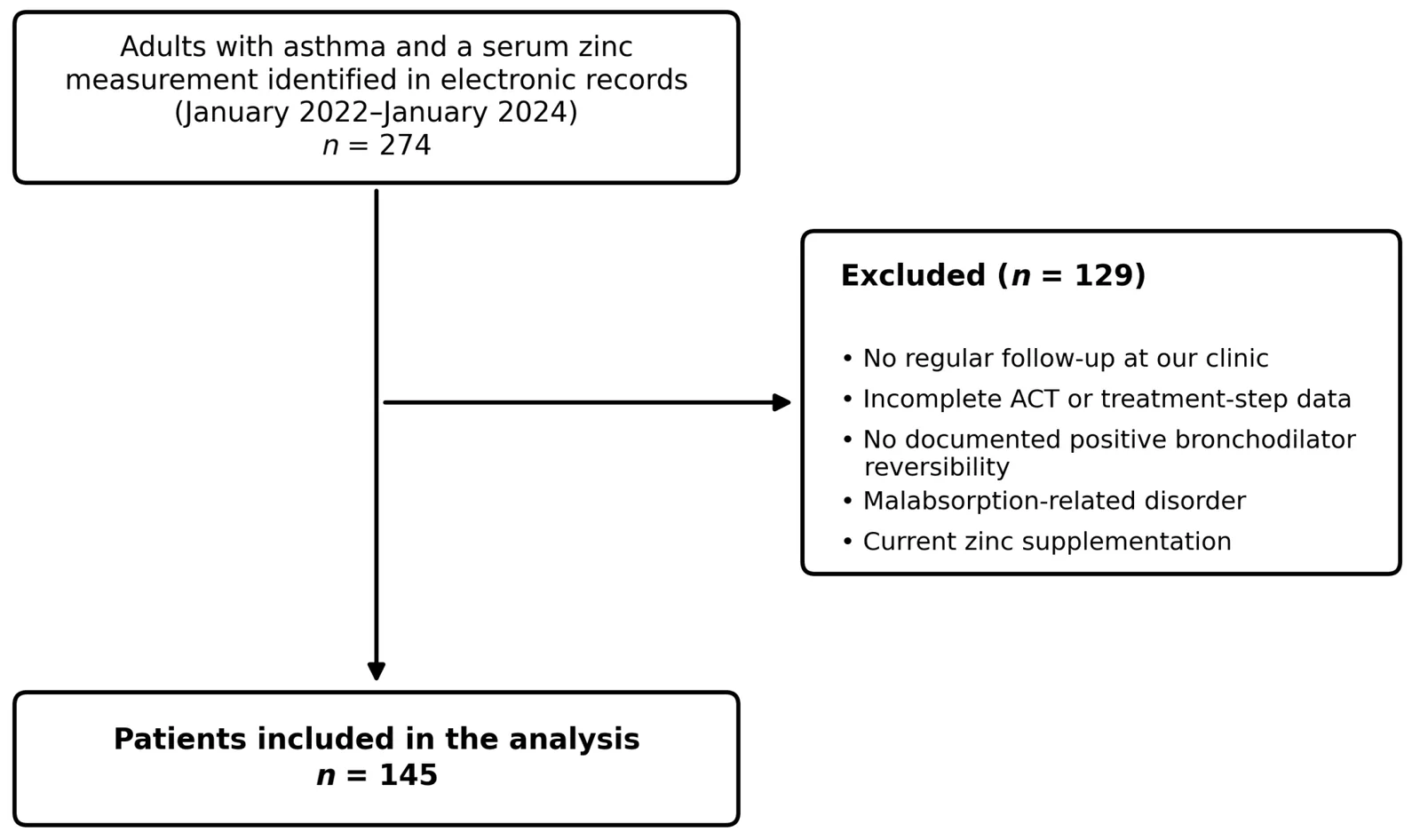
Flow diagram of patient selection. ACT, Asthma Control Test.

The visit at which serum zinc was measured was defined as the index visit. In our clinic, serum zinc is part of the routine laboratory work-up of patients with asthma, so testing was not prompted by suspected nutritional deficiency or other clinical concerns. For patients with more than one measurement, we used the one obtained at the visit at which the ACT score and the other study variables had been recorded. All patients meeting the eligibility criteria during the study period were included. Patients were eligible if an Asthma Control Test (ACT) score, current asthma treatment information, and the clinical variables required for the planned analyses were available for this visit. Each patient contributed one observation to the analysis. Of the 274 adults with asthma and a serum zinc measurement identified during the study period, 129 did not meet the eligibility criteria (no regular follow-up at our clinic, incomplete ACT or treatment-step data, no documented positive bronchodilator reversibility, a malabsorption-related disorder, or current zinc supplementation), leaving 145 patients for analysis (Figure 1). As the study was designed to investigate differences in serum zinc concentrations within an adult asthma cohort, a healthy control group was not included.

### 2.2. Data Collection and Clinical Assessments

Extracted variables comprised age, sex, body mass index (BMI), smoking status, age at asthma onset, asthma duration, allergic rhinitis, serum zinc concentration, C-reactive protein (CRP), blood eosinophil count, total serum immunoglobulin E (IgE), and spirometric indices (forced expiratory volume in 1 s [FEV1], forced vital capacity [FVC], and the FEV1/FVC ratio). The ACT score, asthma treatment step, and biologic treatment status recorded at the index visit were used in the analyses. Serum zinc, CRP, the ACT score, and spirometry were all obtained at the index visit.

The five-item ACT captures the patient’s experience over the previous four weeks, including activity limitation, dyspnea, nocturnal symptoms, rescue-medication use, and perceived overall control. For analysis, scores > 19 indicated controlled asthma and scores ≤ 19 indicated uncontrolled asthma [1].

For this study, asthma severity was operationalized from the treatment step documented at the index visit, in accordance with the 2022 update issued jointly by the Turkish Thoracic Society and the Turkish National Society of Allergy and Clinical Immunology [13]. Steps 1–2, step 3, and steps 4–5 represented mild, moderate, and severe asthma, respectively. This treatment-step-based classification was evaluated separately from ACT-defined current symptom control. Treatment adherence and inhaler technique are checked at every visit in our clinic. Even so, the categories were assigned retrospectively from the prescribed treatment step and therefore reflect treatment intensity rather than an adjudicated severe asthma phenotype; we refer to them as treatment-step categories (steps 1–2, step 3, and steps 4–5) throughout.

Asthma-related healthcare use and systemic corticosteroid exposure were evaluated during the 12 months preceding the index visit. Asthma-related hospitalization and emergency department attendance were recorded as binary variables according to whether at least one documented event had occurred during this period. Systemic corticosteroid exposure was similarly categorized according to whether at least one treatment course had been documented during the preceding 12 months. Patients receiving omalizumab or mepolizumab at the index visit were categorized as receiving biologic treatment (omalizumab, *n* = 35; mepolizumab, *n* = 5).

### 2.3. Exclusion Criteria

Patients were excluded if they had no regular follow-up at our clinic, incomplete clinical records that prevented determination of the ACT-based asthma-control category or treatment-step category at the index visit, a documented disorder associated with malabsorption, or current zinc supplementation.

### 2.4. Measurement of Serum Zinc

The institution’s central biochemistry laboratory quantified serum zinc photometrically by the Nitro-PAPS method, using an Archem Diagnostics reagent kit (Istanbul, Türkiye) and an Abbott ARCHITECT c16000 automated analyzer (Abbott Laboratories, Abbott Park, IL, USA).

According to the manufacturer’s performance data, the repeatability coefficients of variation were 1.61% and 2.55% at zinc concentrations of 95 and 136 µg/dL, respectively. The corresponding within-laboratory precision coefficients of variation were 3.26% and 2.35%. The laboratory reference interval was 70–114 µg/dL.

Blood samples were drawn in the fasting state before noon. The laboratory flags hemolyzed samples, and none of the included samples was hemolyzed. CRP was measured by immunoturbidimetry on the same analyzer (reference range 0–5 mg/L); values reported as below 2 mg/L were assigned a value of 2 mg/L.

Analyses treated serum zinc as a continuous measurement and as a category based on the laboratory’s lower reference limit. Values < 70 µg/dL were considered below the reference range, while values ≥ 70 µg/dL were grouped as within or above that range.

### 2.5. Ethical Approval

The study was conducted in accordance with the Declaration of Helsinki and was approved by the Bursa Uludağ University Faculty of Medicine Clinical Research Ethics Committee on 24 April 2024 (decision no. 2024-66). Under the ethics committee decision, written informed consent was not required for this retrospective review of existing records.

### 2.6. Statistical Analysis

Analyses were performed with IBM SPSS Statistics, version 20.0 (IBM Corp., Armonk, NY, USA). Normality was assessed with the Shapiro–Wilk test and by visual inspection of histograms and Q–Q plots; serum zinc was approximately normally distributed (*p* = 0.71). Approximately normal variables are summarized as mean ± standard deviation and skewed variables (CRP, blood eosinophil count, total IgE, asthma duration, and spirometric indices) as median (interquartile range, IQR); categorical variables are given as counts and percentages.

Two-group comparisons used Student’s *t*-test or the Mann–Whitney U test, and comparisons across the three treatment-step categories used one-way analysis of variance (ANOVA) or the Kruskal–Wallis test, as appropriate. Categorical variables were compared with Pearson’s chi-square test, except for the zinc category, for which Fisher’s exact test was used because few patients had values below 70 µg/dL. Between-group differences in zinc are reported with 95% confidence intervals (CIs). Because age differed across treatment-step categories, that comparison was repeated with adjustment for age by analysis of covariance (ANCOVA). The correlation between zinc and the continuous ACT score was assessed with Pearson’s coefficient.

To examine whether the association between zinc and asthma control was independent of potential confounders, we fitted three sequential models. Linear regression provided the adjusted mean difference in zinc between controlled and uncontrolled asthma; logistic regression provided the odds of uncontrolled asthma per 10 µg/dL higher zinc and for zinc below 70 µg/dL; and linear regression with the ACT score as a continuous outcome estimated the change in ACT per 10 µg/dL of zinc, avoiding the information lost by dichotomizing the score. Model 1 included age and sex, Model 2 added CRP, and Model 3 further added smoking status and treatment-step category. Covariates were chosen on clinical grounds for their known or plausible relationships with circulating zinc and asthma control, and their number was kept small (no more than seven parameters for 59 patients with uncontrolled asthma) to limit overfitting. CRP was log-transformed. Because treatment step is closely tied to asthma control, adjusting for it may remove part of the association of interest, so Model 3 should be read as a conservative estimate. BMI was missing for 22 patients (15.2%) and was therefore examined in a sensitivity analysis restricted to the 123 patients with complete data. Multicollinearity was checked with variance inflation factors. Complete spirometry (FEV1, FVC, and FEV1/FVC) was available for 112 patients (77.2%); FEV1 was added to Model 3 in a second sensitivity analysis restricted to these patients. The relationships of zinc with FEV1 and FEV1/FVC were examined with Spearman’s correlation coefficient and by linear regression adjusted for age and sex.

Missing data, which affected only BMI and spirometry, were not imputed; analyses involving these variables were restricted to patients with available data. The comparison of zinc between controlled and uncontrolled asthma was the primary analysis; comparisons by treatment-step category, healthcare use, systemic corticosteroid exposure, and biologic treatment were exploratory. We did not adjust for multiple comparisons and interpret these secondary results as hypothesis-generating, placing more weight on effect estimates and their CIs than on *p* values. All tests were two-sided, and *p* < 0.05 was considered statistically significant.

## 3. Results

The patient selection process is shown in Figure 1. The analytic cohort comprised 145 adults with asthma. Table 1 summarizes the cohort’s baseline demographic and clinical profile. Participants had a mean age of 46.0 ± 15.6 years; 116 (80.0%) were women, and 46 (31.7%) had a smoking history. The mean age at asthma onset was 36.9 ± 14.3 years, and allergic rhinitis was documented in 128 patients (88.3%). According to the treatment-step-based classification, 19 patients (13.1%) were treated at steps 1–2, 49 (33.8%) at step 3, and 77 (53.1%) at steps 4–5. Forty patients (27.6%), all at steps 4–5, were receiving biologic treatment. The mean ACT score was 19.3 ± 4.0, with 86 patients (59.3%) classified as controlled and 59 (40.7%) as uncontrolled. The mean serum zinc concentration in the overall study population was 88.4 ± 17.2 µg/dL, and 16 patients (11.0%) had concentrations below 70 µg/dL.

### 3.1. Serum Zinc and Asthma Control

**Figure 2:**
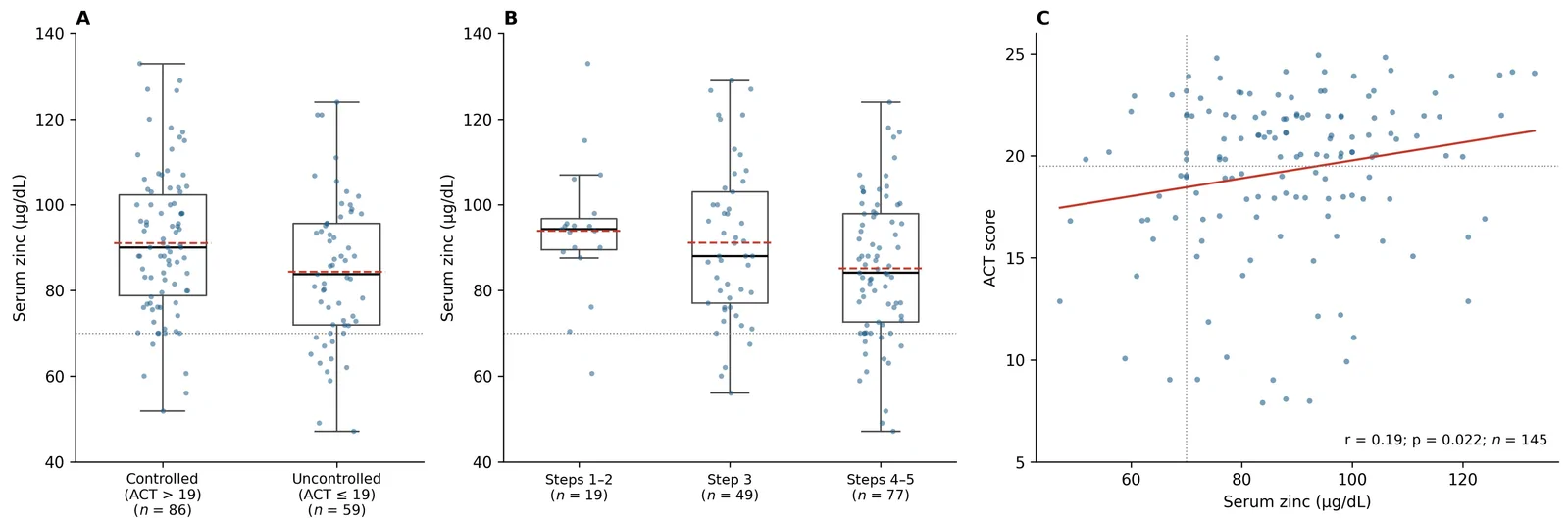
Serum zinc concentrations according to (**A**) ACT-defined asthma control and (**B**) treatment-step-based severity category, and (**C**) relationship between serum zinc and the ACT score (*n* = 145). In (**A**,**B**), boxes show the median and interquartile range, whiskers extend to the most extreme values within 1.5 × the interquartile range, points represent individual patients (with horizontal jitter), and red dashed lines indicate group means. Dotted lines indicate the lower limit of the laboratory reference interval (70 µg/dL) and, in (**C**), the ACT cut-off between controlled and uncontrolled asthma. In (**C**), the solid line is the least-squares regression line; a small vertical jitter was applied to ACT scores to reduce overlap. ACT, Asthma Control Test.

Mean serum zinc was higher in controlled than uncontrolled asthma (91.1 ± 17.0 vs. 84.4 ± 16.9 µg/dL; mean difference 6.7 µg/dL, 95% CI 1.0 to 12.3; *p* = 0.021; Table 2; Figure 2A). The difference was modest, however, and the mean value in each group remained within the laboratory reference interval. The controlled and uncontrolled groups did not differ significantly in age (44.7 ± 15.5 vs. 47.9 ± 15.6 years; *p* = 0.231), BMI (26.5 ± 4.0 vs. 25.7 ± 4.1 kg/m^2^; *p* = 0.274), smoking history (*p* = 0.918), or serum CRP (median 2.0 [IQR 2.0–2.1] vs. 2.0 [2.0–2.8] mg/L; *p* = 0.450). Sex distribution was also comparable between the groups (*p* = 0.237). Blood eosinophil count, total IgE, FEV1, and FEV1/FVC were similar in the two groups. As expected, uncontrolled asthma was more frequent at higher treatment steps (*p* < 0.001; Table 2). Serum zinc showed a weak positive correlation with the continuous ACT score (*r* = 0.19; *p* = 0.022; Figure 2C). Serum zinc also correlated weakly with FEV1 (Spearman’s ρ = 0.29; *p* = 0.002; *n* = 112) and FEV1/FVC (ρ = 0.25; *p* = 0.008; *n* = 112). After adjustment for age and sex, the association with FEV1 was no longer statistically significant (30 mL per 10 µg/dL higher zinc, 95% CI −35 to 95; *p* = 0.363), whereas the association with FEV1/FVC persisted but was small and imprecise (1.2 percentage points per 10 µg/dL, 95% CI 0.1 to 2.3; *p* = 0.032), with a lower confidence limit close to zero.

### 3.2. Serum Zinc and Treatment-Step-Based Asthma Severity

Mean age increased across the steps 1–2, step 3, and steps 4–5 categories (38.5 ± 11.2, 42.4 ± 15.3, and 50.2 ± 15.6 years, respectively; *p* = 0.002). Sex distribution, BMI, and serum CRP were comparable across the three categories (*p* = 0.528, *p* = 0.212, and *p* = 0.309, respectively). The ACT score decreased across the categories (*p* < 0.001).

Mean serum zinc concentrations were 94.0 ± 15.6 µg/dL in patients at steps 1–2, 91.2 ± 18.3 µg/dL in those at step 3, and 85.2 ± 16.4 µg/dL in those at steps 4–5 (Figure 2B). In the unadjusted comparison, the difference across treatment-step categories was of borderline statistical significance (one-way ANOVA, *p* = 0.0497; Table 3). Age increased across the categories and correlated inversely with zinc (*r* = −0.27; *p* < 0.001); after adjustment for age, the difference across treatment-step categories was no longer statistically significant (ANCOVA, *p* = 0.277). Given the exploratory nature of this comparison, we did not perform pairwise tests.

### 3.3. Laboratory-Based Serum Zinc Category and Asthma Control

When serum zinc was categorized according to the lower limit of the laboratory reference interval, 16 patients (11.0%) had concentrations below 70 µg/dL and 129 (89.0%) had concentrations of 70 µg/dL or higher. Zinc below 70 µg/dL was more frequent in uncontrolled than in controlled asthma (11/59 [18.6%] vs. 5/86 [5.8%]; odds ratio [OR] for uncontrolled asthma 3.71, 95% CI 1.22 to 11.33; Fisher’s exact test, *p* = 0.028; Table 2). With only 16 patients below the cut-off, however, this estimate is imprecise.

### 3.4. Serum Zinc, Healthcare Use, and Treatment Exposure

Mean serum zinc concentration was 81.9 ± 21.0 µg/dL among patients with at least one asthma-related hospitalization during the preceding 12 months and 88.7 ± 17.0 µg/dL among those without hospitalization (*p* = 0.314). The corresponding concentrations were 84.3 ± 18.6 and 89.1 ± 16.9 µg/dL among patients with and without an emergency department visit (*p* = 0.228), and 82.7 ± 18.0 and 89.7 ± 16.8 µg/dL among those with and without systemic corticosteroid exposure (*p* = 0.056), respectively. Patients receiving biologic treatment at the index visit had a mean serum zinc concentration of 84.2 ± 15.8 µg/dL, compared with 90.0 ± 17.5 µg/dL in those not receiving biologic treatment (*p* = 0.069). None of these differences reached statistical significance. The confidence intervals were wide, however, particularly for hospitalization (mean difference −6.7 µg/dL, 95% CI −19.9 to 6.4; *n* = 7), and do not exclude modest differences (Table 4).

### 3.5. Multivariable Analyses

Table 5 shows the sequential models. Adjustment for age and sex reduced the mean difference in zinc between controlled and uncontrolled asthma from 6.7 to 5.2 µg/dL (95% CI −0.2 to 10.6; *p* = 0.061). Adding CRP reduced it further to 4.4 µg/dL (95% CI −1.0 to 9.7; *p* = 0.109), and adding smoking status and treatment step reduced it to 3.1 µg/dL (95% CI −2.6 to 8.8; *p* = 0.281). The odds of uncontrolled asthma per 10 µg/dL of zinc and the continuous ACT score followed the same pattern. The association with zinc below 70 µg/dL remained statistically significant after adjustment for age and sex (OR 3.33, 95% CI 1.08 to 10.31) but not after CRP was added. In the fully adjusted model, female sex (β = −9.2 µg/dL, 95% CI −16.1 to −2.3; *p* = 0.009) and higher log-transformed CRP (β = −5.2 µg/dL per log unit, 95% CI −8.7 to −1.6; *p* = 0.005) were independently associated with lower zinc. Including BMI in the 123 patients with complete data did not materially change the estimates, and all variance inflation factors were below 3. In the 112 patients with complete spirometry, the fully adjusted mean difference was 1.9 µg/dL (95% CI −4.4 to 8.2) and changed little after further adjustment for FEV1 (1.5 µg/dL, 95% CI −5.0 to 7.9; *p* = 0.653; Table 5).

## 4. Discussion

In this retrospective cohort of adults with asthma, serum zinc was lower in patients with uncontrolled asthma, and concentrations below the reference limit were more frequent in this group, findings that resemble those reported in children [11,14]. After adjustment for age, sex, CRP, smoking, and treatment step, however, the association was attenuated and was no longer statistically significant, although a modest difference in favor of controlled asthma could not be excluded. A similar pattern was observed for treatment-step-based severity, for which the difference across categories was no longer statistically significant after adjustment for age. These findings suggest that demographic, inflammatory, and treatment-related factors contribute to the observed association; however, residual confounding and limited precision preclude definitive conclusions.

The size of the unadjusted difference also deserves comment. A gap of 6.7 µg/dL corresponds to roughly 0.4 standard deviations of the zinc distribution in our cohort, and both group means lay within the reference interval; most patients with uncontrolled asthma did not, in fact, have low zinc by laboratory criteria. The continuous and categorical analyses should be read together. Dichotomizing a continuous measurement discards information and statistical power, and our categorical estimate rested on only 16 patients below 70 µg/dL, which explains its wide confidence interval. The direction of the association was consistent across the continuous, categorical, and ACT-based analyses. It is also worth remembering that a reference interval describes the distribution of values in a reference population; it is not a functional threshold below which immune or antioxidant effects become apparent.

The attenuation we observed is consistent with a contribution from factors known to influence serum zinc independently of asthma. Population data consistently show lower serum zinc in women than in men, which is why sex-specific cut-offs have been recommended [7]; in our fully adjusted model, female sex was independently associated with lower zinc, and women were slightly more common in the uncontrolled group. Serum zinc also varies with age, as it did in our cohort, and falls during inflammation, when zinc is redistributed from plasma to tissues as part of the acute-phase response [7]. The inverse association between CRP and zinc in our models is consistent with this effect, although most CRP values were at the lower reporting limit of the assay, and this observation should not be overinterpreted. These observations reinforce the point that serum zinc reflects a circulating concentration sensitive to non-nutritional influences rather than whole-body zinc status [7]. Treatment step was the covariate most strongly related to asthma control; because treatment intensity and disease control are closely linked, the fully adjusted model may remove part of the association of interest and should be regarded as conservative.

Rajkumar et al. prospectively assessed 67 children and adolescents and likewise found that higher zinc concentrations accompanied better asthma symptom control [11]. That study did not, however, define a clear low-zinc cut-off associated with inadequate control. In our cohort, zinc below 70 µg/dL was more common in uncontrolled asthma in the crude analysis, but the estimate was imprecise and was no longer statistically significant after full adjustment. Srivastava et al. observed lower serum zinc concentrations in children with asthma than in healthy controls and reported progressively higher concentrations across uncontrolled, partly controlled, and well-controlled asthma groups [14]. Chen et al. likewise reported lower circulating zinc in asthma in their meta-analysis, a difference that was evident in adult but not in pediatric studies [9]. More recently, Xue et al. synthesized 21 pediatric studies encompassing 2205 participants and found lower circulating zinc in children with asthma, whereas the pooled difference for wheezing alone was not statistically significant [10]. They noted that disease-related reductions in circulating zinc might overstate zinc deficiency and recommended longitudinal evaluation across remission and varying disease activity [10].

A recent review of zinc in pediatric asthma reached a similar overall conclusion: observational studies frequently reported lower zinc concentrations or poorer control in association with lower zinc, whereas interventional findings remained inconsistent and were limited by differences in baseline zinc status, supplementation protocols, and outcome definitions [15]. Because most available studies have involved children and adolescents, their findings cannot be directly generalized to adults. This caveat applies with particular force to our cohort, which was predominantly female (80%), recruited from a tertiary referral center, and weighted toward step 4–5 treatment; zinc requirements, growth-related influences, and asthma phenotypes in children differ considerably from those in such a population. The present study therefore contributes to the comparatively limited adult literature by examining symptom control and treatment-defined severity as separate clinical domains and by testing whether the association with control holds after adjustment for potential confounders.

Our data do not identify the mechanism linking zinc with asthma control. More active inflammation could lower circulating zinc through inflammation-related redistribution. Reverse causation is therefore entirely plausible: lower circulating zinc may accompany poor control or heightened inflammatory activity rather than precede it. In the opposite direction, inadequate zinc availability could weaken antioxidant capacity and immune regulation, thereby intensifying inflammatory responses [16]. The observed difference may therefore reflect an effect of inflammatory activity, a factor contributing to poor control, or reciprocal influences. In our models, adjustment for CRP accounted for part of the attenuation of the association with control; CRP, however, is a systemic marker and does not directly reflect airway inflammation. These competing explanations cannot be separated in a retrospective dataset containing one zinc measurement per patient.

Recent reviews likewise emphasize that zinc-related immune effects depend on tightly regulated cellular distribution and that inflammatory responses may themselves alter circulating zinc concentrations [6,12]. This biological complexity supports interpreting serum zinc as an associated marker in the present study rather than evidence of a single directional pathway.

Interventional evidence remains sparse. In one randomized study, 42 children hospitalized with an asthma exacerbation received either zinc bisglycinate providing 30 mg elemental zinc daily or placebo in addition to standard exacerbation therapy. The zinc-treated group showed a greater reduction in exacerbation severity at 24 and 48 h [17]. Because the trial was small, pediatric, and conducted during acute exacerbations, its results are not directly applicable to stable adult asthma. A recent narrative review concluded that the available evidence does not support routine trace-element assessment or generalized supplementation in allergic disease outside documented deficiency or a clearly defined clinical indication [12].

Numerically, mean zinc concentrations were greater at steps 1–2 and step 3 than at steps 4–5. However, this difference did not persist after adjustment for age, which differed markedly across categories and correlated inversely with zinc. This comparison therefore provides no clear evidence of a relationship between serum zinc and treatment-step-based severity, although a small difference cannot be excluded. Kuti et al. reported lower zinc concentrations among children with asthma than among healthy controls but found no association of zinc with either asthma severity or symptom control [18]. Khanbabaee et al. evaluated 100 children with asthma against a healthy control group and documented lower mean zinc in the asthma group together with an association between zinc status and asthma severity. Serum zinc was not related to disease control in that study [19].

Defining severity by treatment step requires some caution. Treatment steps are shaped by prescribing habits, adherence, comorbidities, drug availability, and cost, and they do not fully capture intrinsic disease severity [1]. Adherence and inhaler technique are reviewed routinely in our clinic, but severity was still assigned retrospectively from the prescribed treatment, and some misclassification is likely.

Blood eosinophil count, total IgE, FEV1, and FEV1/FVC did not differ significantly between the control groups (Table 2). Zinc correlated weakly with FEV1 and FEV1/FVC. After adjustment for age and sex, only the association with FEV1/FVC persisted; it was small and imprecisely estimated and, given the number of exploratory comparisons, should be interpreted with caution. Adding FEV1 to the fully adjusted model did not materially change the association between zinc and asthma control. Because FeNO was not available, complete spirometry was missing in 22.8% of patients, and exacerbations were recorded only as binary events over the preceding year, the relationship between zinc and objective measures of airway inflammation and lung function remains to be fully characterized.

Ghaffari et al., in another meta-analysis, detected neither a difference in mean zinc between children with and without asthma nor an association with asthma severity [20]. Variation in design, participant characteristics, current disease activity, diet, genetic background, sampling schedule, and laboratory methodology may account for the inconsistent literature. Future longitudinal work should standardize zinc assessment across different phases of asthma and incorporate dietary exposure and treatment-related covariates.

A recent Mendelian randomization study linked genetically predicted higher circulating zinc to a modestly lower asthma risk [21]. Although this finding adds biological plausibility, it concerns susceptibility to asthma rather than symptom control or severity among patients with established disease. It therefore does not confirm causality for the association observed in our cohort or establish that increasing zinc concentrations would improve asthma outcomes.

In our cohort, zinc did not vary significantly by hospitalization, emergency department use, systemic corticosteroid exposure, or biologic treatment (Table 4). Because all patients receiving biologics were in the steps 4–5 treatment-step category, this comparison cannot be separated from treatment intensity. Adult evidence addressing zinc in relation to these outcomes is scarce. In one small study of adults, serum zinc was lower during acute exacerbations than after inhaled corticosteroid treatment and lower than in asymptomatic patients, suggesting that circulating zinc fluctuates with disease activity [22]; a single measurement obtained at a routine visit may therefore not reflect concentrations at the time of these events. The hospitalization subgroup was particularly small, and the other exploratory comparisons may also have been underpowered to detect modest differences. Larger adult studies with multivariable adjustment should assess whether zinc is independently related to exacerbation outcomes or treatment intensity.

Taken together, the attenuation of the association after adjustment, particularly for CRP, is biologically plausible. Zinc behaves as a negative acute-phase reactant: in experimental models, interleukin-6 increases hepatic zinc uptake, shifting zinc from the circulation to the liver and lowering serum concentrations independently of body zinc stores [23]. In large population surveys, serum zinc concentrations were lower at higher concentrations of inflammatory markers such as CRP, and adjustment for inflammation has been proposed when zinc status is assessed [24]. Lower serum zinc in uncontrolled asthma may therefore partly reflect inflammatory activity. Whether low zinc also contributes to inflammation cannot be determined in a cross-sectional design; if it does, adjustment for CRP may have removed part of a true effect rather than only confounding.

### Limitations

Some limitations should be considered when interpreting these findings. The retrospective, single-center, cross-sectional design does not allow us to determine whether low zinc precedes or follows poor control, and residual confounding by unmeasured factors cannot be excluded despite adjustment. Serum zinc was measured once. Samples were drawn in the fasting state before noon and none was hemolyzed, but circulating zinc is also affected by the acute-phase response, stress, recent dietary intake, and diurnal variation [7], and the retrospective design did not allow us to confirm that every patient was free of an exacerbation or respiratory infection at the time of sampling. A single value may therefore not reflect long-term zinc status or concentrations during the hospitalizations, emergency visits, or corticosteroid courses of the preceding year.

Information on dietary zinc intake, supplements other than zinc, other trace elements, and FeNO was not available, and socioeconomic data were incompletely recorded and were therefore not included in the analyses; complete spirometry was unavailable for 33 patients (22.8%), although these patients did not differ significantly from those with complete spirometry in age, sex, serum zinc, ACT score, CRP, or treatment step. Because severity was inferred from treatment step, some patients may have been misclassified, and without a healthy control group we could not determine whether adults with asthma have lower zinc than adults without asthma. Zinc is measured routinely in patients with asthma in our clinic, which makes selective testing unlikely; nevertheless, nearly half of the 274 screened patients were excluded and some selection bias cannot be ruled out. The cohort was predominantly female, drawn from a tertiary referral center, and weighted toward higher treatment steps, which limits generalizability to primary care and milder disease. Finally, some subgroups were small, notably hospitalization (*n* = 7) and zinc below 70 µg/dL (*n* = 16). These estimates were imprecise, and the nonsignificant results should not be interpreted as establishing the absence of an association. Prospective studies with repeated, standardized zinc measurements and validated dietary assessment would address many of these issues.

## 5. Conclusions

In adults with asthma, higher serum zinc concentrations were associated with controlled asthma in unadjusted analyses; however, the association was attenuated after adjustment for age, sex, CRP, smoking, and treatment step, and mean concentrations in both groups remained within the reference interval. Differences across treatment-step categories were not statistically significant after adjustment for age, and exploratory comparisons by healthcare use and treatment exposure were imprecise. These findings suggest that serum zinc in asthma may partly reflect inflammatory status, although the direction of this relationship remains unresolved; they do not establish an independent or causal relationship, and whether zinc supplementation improves asthma control requires further study. A low serum zinc concentration in a patient with poorly controlled asthma should therefore be interpreted together with inflammatory and nutritional status. Longitudinal studies with repeated, inflammation-adjusted zinc measurements and dietary assessment, as well as randomized trials of zinc repletion in patients with documented deficiency, are needed to determine whether serum zinc is a modifiable contributor to asthma control, a marker of inflammation, or both.

## Figures and Tables

**Table 1:** Baseline demographic and clinical characteristics of the study population.

Characteristic	Overall Population (*n* = 145)
Age, years	46.0 ± 15.6
Sex, *n* (%)	
Female	116 (80.0)
Male	29 (20.0)
BMI, kg/m^2^	26.1 ± 4.1
Smoking history, *n* (%)	46 (31.7)
Age at asthma onset, years	36.9 ± 14.3
Asthma duration, years	7 (3–12)
Allergic rhinitis, *n* (%)	128 (88.3)
Treatment-step category, *n* (%)	
Steps 1–2	19 (13.1)
Step 3	49 (33.8)
Steps 4–5	77 (53.1)
Biologic treatment at the index visit, *n* (%)	40 (27.6)
Omalizumab	35 (24.1)
Mepolizumab	5 (3.4)
Asthma-related events in the preceding 12 months, *n* (%)	
Hospitalization	7 (4.8)
Emergency department attendance	22 (15.2)
Systemic corticosteroid course	27 (18.6)
Asthma-control category, *n* (%)	
Controlled (ACT > 19)	86 (59.3)
Uncontrolled (ACT ≤ 19)	59 (40.7)
ACT score	19.3 ± 4.0
Serum zinc, µg/dL	88.4 ± 17.2
Serum zinc < 70 µg/dL, *n* (%)	16 (11.0)
CRP, mg/L	2.0 (2.0–2.2)
CRP > 5 mg/L, *n* (%)	21 (14.5)
Blood eosinophils, cells/µL	175 (94–311)
Total IgE, IU/mL	89 (21–257)
FEV1, mL	2480 (2080–3125)
FEV1/FVC, %	80.5 (76.0–84.0)

Data are presented as mean ± standard deviation, median (interquartile range), or number (percentage), as appropriate. CRP values reported below the lower reporting limit of the assay were assigned a value of 2 mg/L. Data were complete for all variables except BMI (available for 123 patients) and spirometry (FEV1 and FEV1/FVC; 112 patients). ACT, Asthma Control Test; BMI, body mass index; CRP, C-reactive protein; FEV1, forced expiratory volume in 1 s; FVC, forced vital capacity; IgE, immunoglobulin E.

**Table 2:** Clinical and laboratory characteristics according to ACT-defined asthma-control status.

Characteristic	Controlled Asthma, ACT > 19 (*n* = 86)	Uncontrolled Asthma, ACT ≤ 19 (*n* = 59)	*p*
Age, years	44.7 ± 15.5	47.9 ± 15.6	0.231 ^a^
Female sex, *n* (%)	66 (76.7)	50 (84.7)	0.237 ^c^
BMI, kg/m^2^ (*n* = 71/52)	26.5 ± 4.0	25.7 ± 4.1	0.274 ^a^
Smoking history, *n* (%)	27 (31.4)	19 (32.2)	0.918 ^c^
CRP, mg/L	2.0 (2.0–2.1)	2.0 (2.0–2.8)	0.450 ^b^
CRP > 5 mg/L, *n* (%)	9 (10.5)	12 (20.3)	0.097 ^c^
Blood eosinophils, cells/µL	206 (120–311)	150 (79–298)	0.173 ^b^
Total IgE, IU/mL	98 (22–259)	72 (22–212)	0.891 ^b^
FEV1, mL (*n* = 63/49)	2560 (2210–3240)	2400 (1990–2910)	0.085 ^b^
FEV1/FVC, % (*n* = 63/49)	81.0 (76.0–84.0)	80.0 (76.0–84.0)	0.953 ^b^
Treatment-step category, *n* (%)			**<0.001** ^c^
Steps 1–2	18 (20.9)	1 (1.7)	
Step 3	32 (37.2)	17 (28.8)	
Steps 4–5	36 (41.9)	41 (69.5)	
Serum zinc, µg/dL	91.1 ± 17.0	84.4 ± 16.9	**0.021** ^a^
Mean difference in zinc (95% CI), µg/dL	6.7 (1.0 to 12.3)	—	
Serum zinc < 70 µg/dL, *n* (%)	5 (5.8)	11 (18.6)	**0.028** ^d^
Serum zinc ≥ 70 µg/dL, *n* (%)	81 (94.2)	48 (81.4)	

Data are presented as mean ± standard deviation, median (interquartile range), or number (percentage), as appropriate. Where data were incomplete, the number of patients with available data is shown (controlled/uncontrolled). ^a^ Student’s *t*-test; ^b^ Mann–Whitney U test; ^c^ Pearson’s chi-square test; ^d^ Fisher’s exact test. ACT, Asthma Control Test; BMI, body mass index; CI, confidence interval; CRP, C-reactive protein; FEV1, forced expiratory volume in 1 s; FVC, forced vital capacity; IgE, immunoglobulin E. Statistically significant *p* values are shown in bold.

**Table 3:** Clinical characteristics and serum zinc concentrations according to treatment-step-based asthma severity.

Characteristic	Steps 1–2 (*n* = 19)	Step 3 (*n* = 49)	Steps 4–5 (*n* = 77)	*p*
Age, years	38.5 ± 11.2	42.4 ± 15.3	50.2 ± 15.6	**0.002** ^a^
Female sex, *n* (%)	17 (89.5)	38 (77.6)	61 (79.2)	0.528 ^c^
BMI, kg/m^2^ (*n* = 16/39/68)	25.4 ± 5.1	25.4 ± 3.2	26.7 ± 4.2	0.212 ^a^
Smoking history, *n* (%)	7 (36.8)	10 (20.4)	29 (37.7)	0.112 ^c^
CRP, mg/L	2.0 (2.0–2.5)	2.0 (2.0–2.0)	2.0 (2.0–3.0)	0.309 ^b^
ACT score	22.8 ± 1.5	19.6 ± 3.8	18.2 ± 3.9	**<0.001** ^a^
Serum zinc, µg/dL	94.0 ± 15.6	91.2 ± 18.3	85.2 ± 16.4	**0.0497** ^a^
Serum zinc, age-adjusted *p*				0.277 ^d^

Data are presented as mean ± standard deviation, median (interquartile range), or number (percentage), as appropriate. ^a^ One-way analysis of variance; ^b^ Kruskal–Wallis test; ^c^ Pearson’s chi-square test; ^d^ analysis of covariance with age as covariate. The *p* value for serum zinc is reported to four decimal places because the rounded value (0.050) lies at the significance threshold. BMI, body mass index; CRP, C-reactive protein. Statistically significant *p* values are shown in bold.

**Table 4:** Serum zinc concentrations according to asthma-related healthcare use, systemic corticosteroid exposure, and biologic treatment.

Variable	Category	Serum Zinc, µg/dL	Mean Difference (95% CI), µg/dL	*p*
Asthma-related hospitalization	Yes (*n* = 7)	81.9 ± 21.0	−6.7 (−19.9 to 6.4)	0.314 ^a^
	No (*n* = 138)	88.7 ± 17.0	—	
Emergency department attendance	Yes (*n* = 22)	84.3 ± 18.6	−4.8 (−12.7 to 3.0)	0.228 ^a^
	No (*n* = 123)	89.1 ± 16.9	—	
Systemic corticosteroid exposure	Yes (*n* = 27)	82.7 ± 18.0	−7.0 (−14.2 to 0.2)	0.056 ^a^
	No (*n* = 118)	89.7 ± 16.8	—	
Biologic treatment at the index visit	Receiving (*n* = 40)	84.2 ± 15.8	−5.8 (−12.1 to 0.5)	0.069 ^a^
	Not receiving (*n* = 105)	90.0 ± 17.5	—	

Data are presented as mean ± standard deviation. Hospitalization, emergency department attendance, and systemic corticosteroid exposure were evaluated during the 12 months preceding the index visit; biologic treatment status was determined at the index visit. ^a^ Student’s *t*-test. Mean differences are calculated as the “Yes/Receiving” group minus the “No/Not receiving” group. All patients receiving biologic treatment belonged to the steps 4–5 treatment-step category. CI, confidence interval.

**Table 5:** Unadjusted and adjusted associations between serum zinc and asthma control.

Model	Mean Difference in Zinc, Controlled vs. Uncontrolled, µg/dL	OR for Uncontrolled Asthma per 10 µg/dL Higher Zinc	OR for Uncontrolled Asthma, Zinc < 70 vs. ≥ 70 µg/dL	Change in ACT Score per 10 µg/dL Higher Zinc
Unadjusted (*n* = 145)	6.7 (1.0 to 12.3) *p* = 0.021	0.79 (0.64–0.97) *p* = 0.024	3.71 (1.22–11.33) *p* = 0.021	0.44 (0.07 to 0.81) *p* = 0.022
Model 1 (*n* = 145)	5.2 (−0.2 to 10.6) *p* = 0.061	0.81 (0.66–1.01) *p* = 0.061	3.33 (1.08–10.31) *p* = 0.037	0.40 (−0.01 to 0.80) *p* = 0.053
Model 2 (*n* = 145)	4.4 (−1.0 to 9.7) *p* = 0.109	0.83 (0.67–1.04) *p* = 0.106	2.98 (0.93–9.58) *p* = 0.066	0.32 (−0.09 to 0.73) *p* = 0.126
Model 3 (*n* = 145)	3.1 (−2.6 to 8.8) *p* = 0.281	0.87 (0.69–1.10) *p* = 0.256	2.79 (0.82–9.56) *p* = 0.102	0.18 (−0.21 to 0.57) *p* = 0.359
Model 3 + BMI (*n* = 123)	2.6 (−3.5 to 8.8) *p* = 0.399	0.90 (0.70–1.16) *p* = 0.403	3.76 (0.84–16.89) *p* = 0.084	0.18 (−0.27 to 0.62) *p* = 0.425
Model 3 + FEV1 (*n* = 112)	1.5 (−5.0 to 7.9) *p* = 0.653	0.94 (0.72–1.22) *p* = 0.643	2.40 (0.60–9.61) *p* = 0.216	0.01 (−0.46 to 0.49) *p* = 0.957

Values are estimates (95% confidence interval). Mean differences and changes in ACT score were estimated by linear regression, and odds ratios by logistic regression. Model 1: adjusted for age and sex. Model 2: Model 1 + log-transformed CRP. Model 3: Model 2 + smoking status and treatment-step category (steps 1–2, step 3, steps 4–5). Model 3 + BMI: sensitivity analysis restricted to patients with available BMI. Model 3 + FEV1: sensitivity analysis restricted to patients with complete spirometry (FEV1 entered in liters). ACT, Asthma Control Test; BMI, body mass index; CRP, C-reactive protein; OR, odds ratio. The *p* values in this table were derived from the regression models (*t*-test for linear regression; Wald test for logistic regression); the unadjusted *p* value for zinc < 70 µg/dL therefore differs from that of Fisher’s exact test in Table 2 (*p* = 0.028).

## Data Availability

The data presented in this study are available on reasonable request from the corresponding author due to patient privacy restrictions.
